# Supplementary figures and images for: Exploring the genetic diversity of the Japanese population: Insights from a large-scale whole genome sequencing analysis
Source: PLoS Genet. 2023 Dec 7;19(12):e1010625. doi: 10.1371/journal.pgen.1010625 (PMC10703243; doi:10.1371/journal.pgen.1010625)

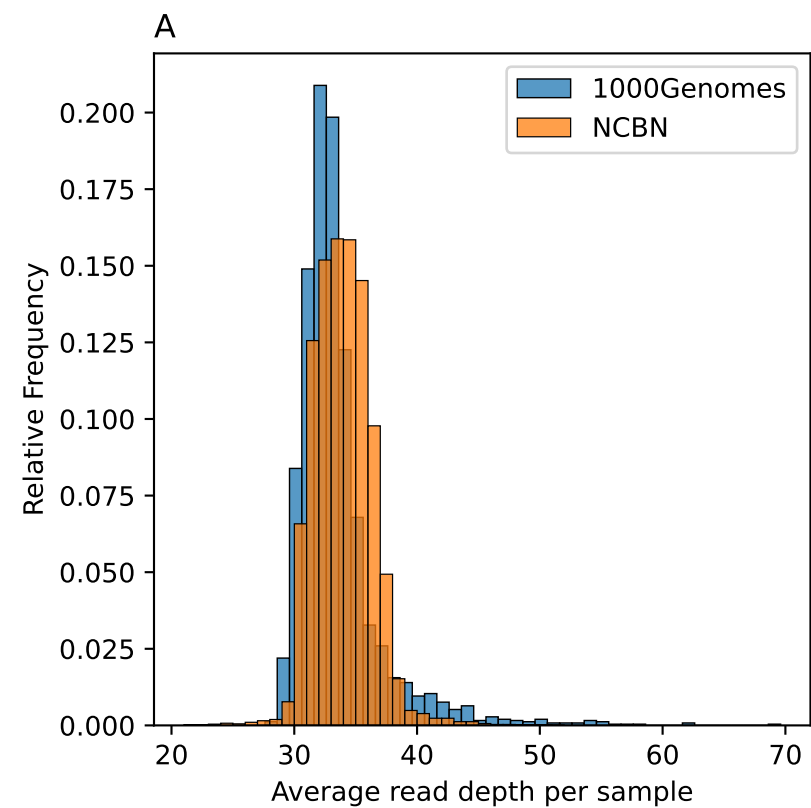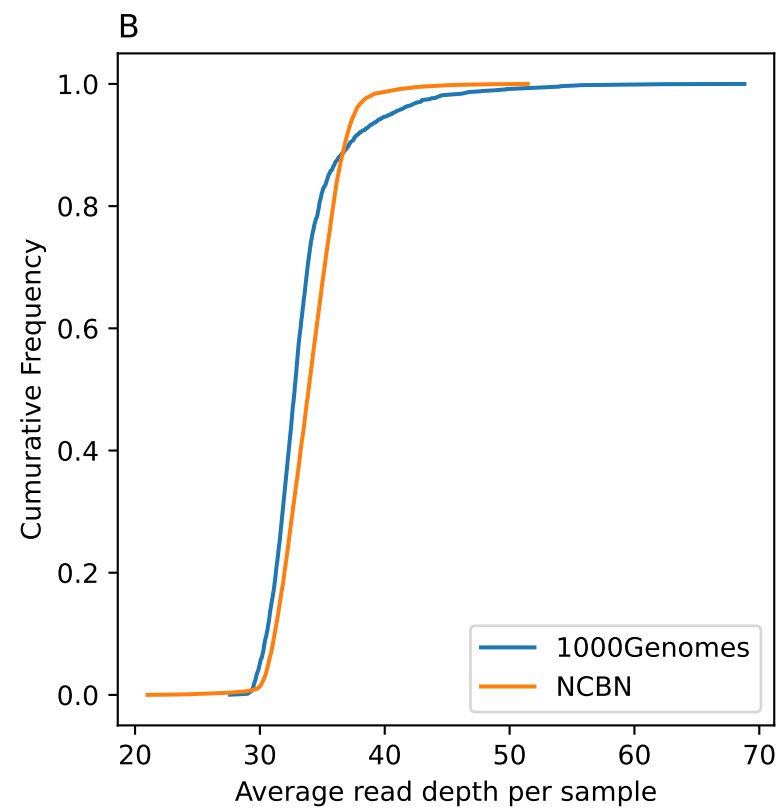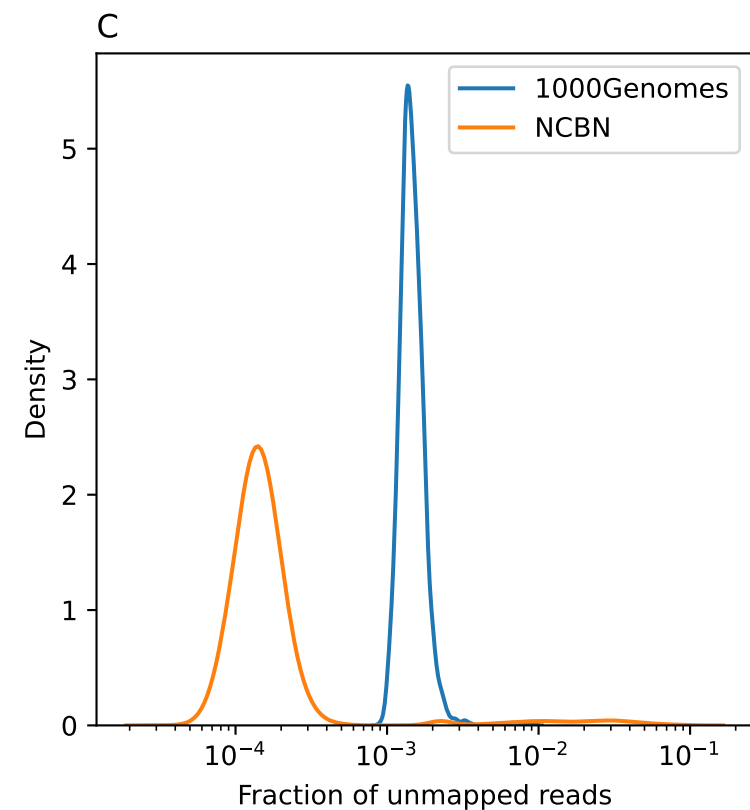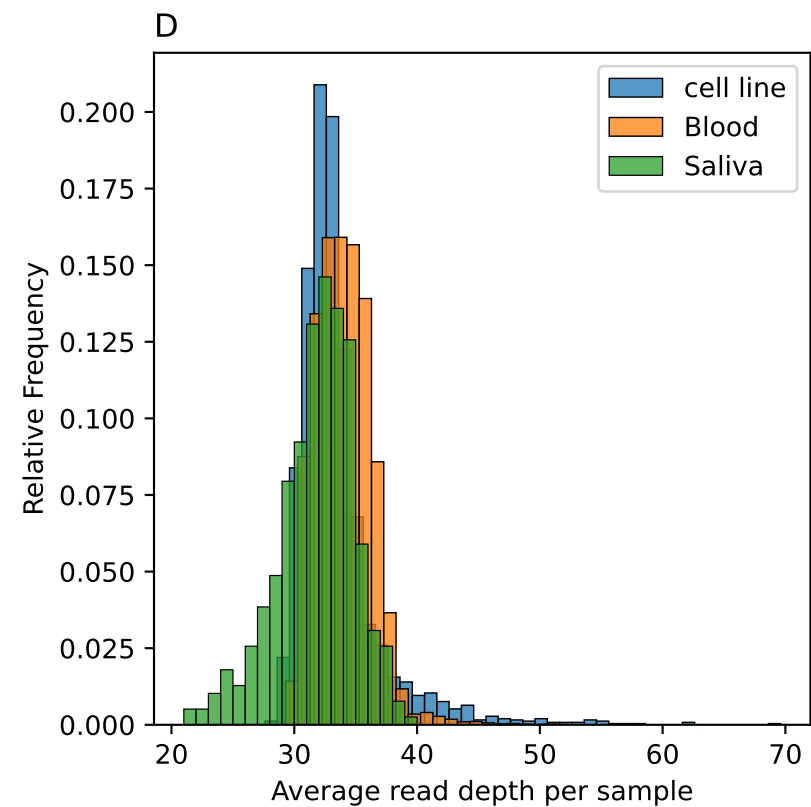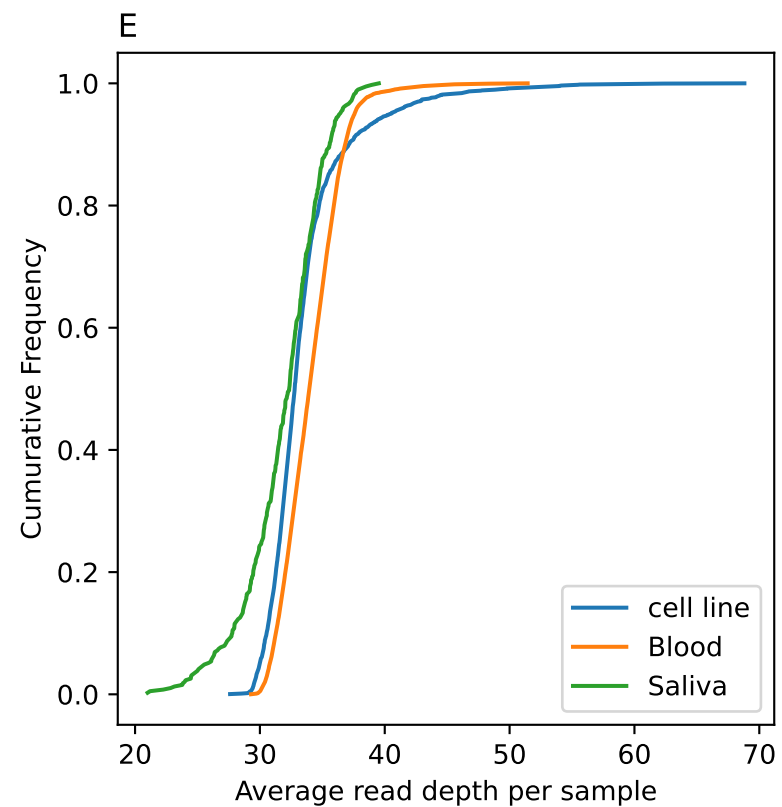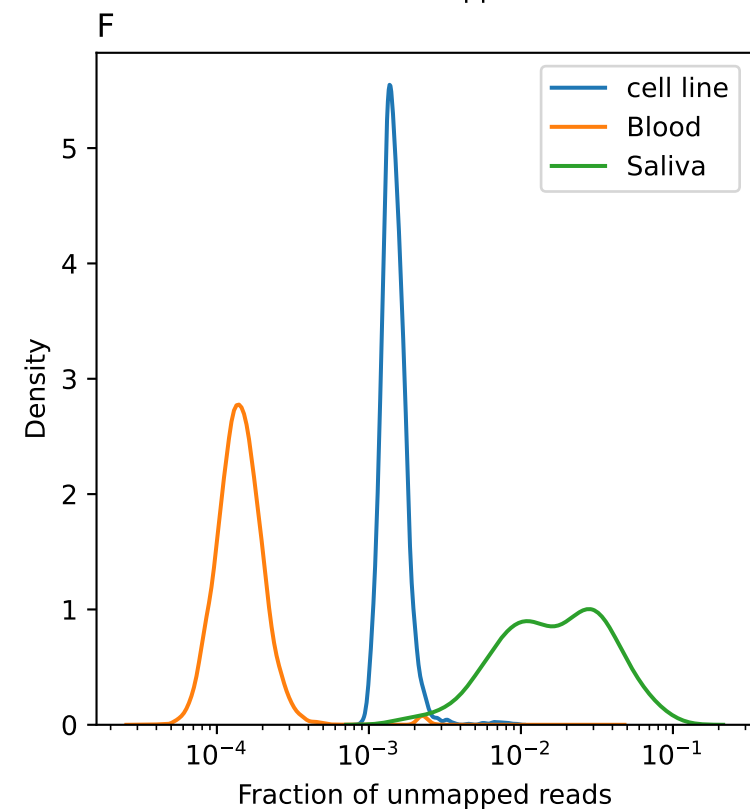

Supplement: S1 Fig — Comparison of QC metrics between NCBN and 1000 Genome samples. (A)(D) Histogram of average depth per sample. (B)(E) Cumulative frequencies of average depth per sample. Comparisons are made between NCBN and 1000Genome samples in (A)(B)(C); Comparisons are made between blood, saliva (both NCBN) and cell line (1000Genomes) in (D)(E)(F). (PDF) [file pgen.1010625.s001.pdf]

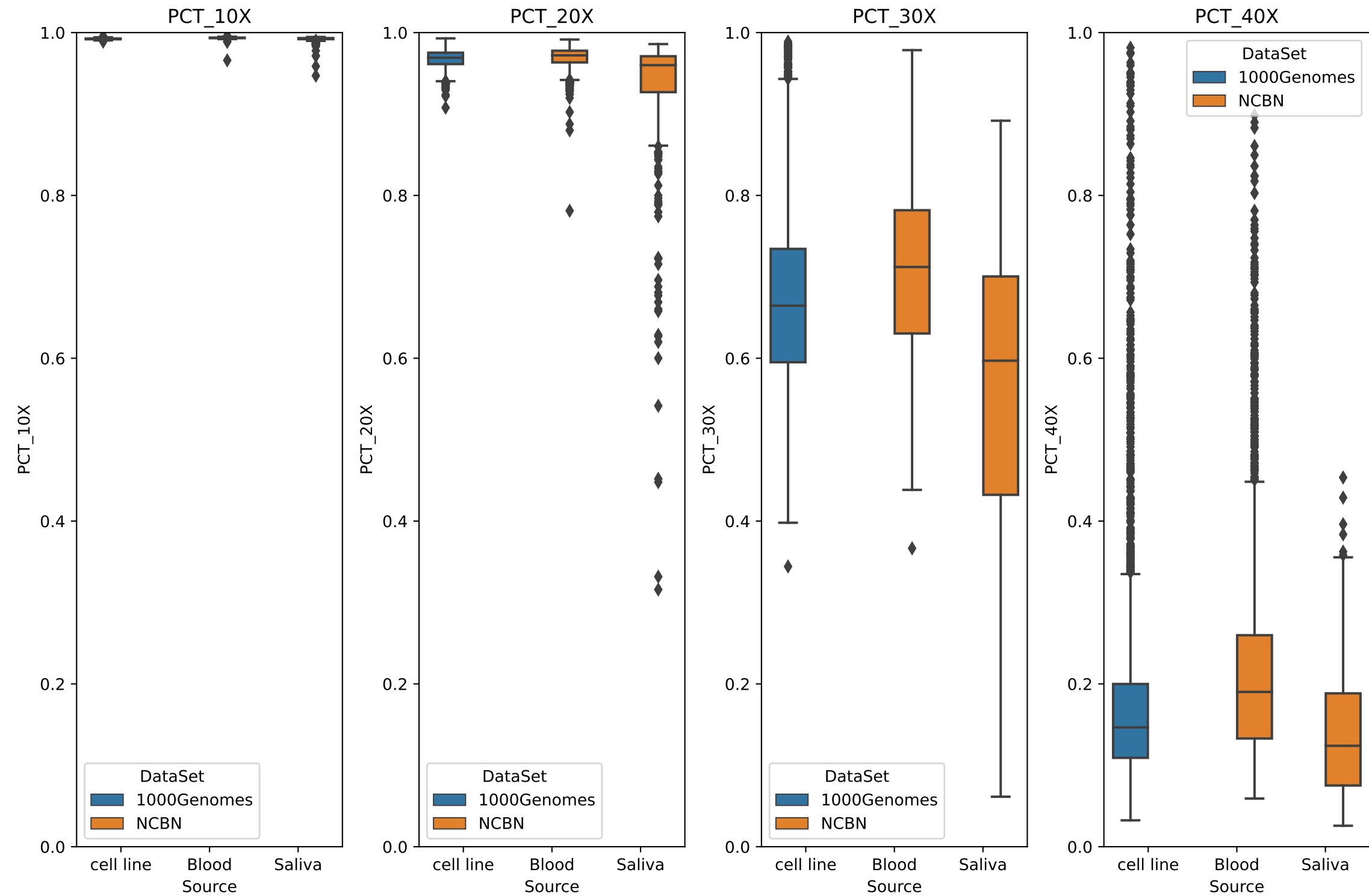

Supplement: S2 Fig — The fraction of genomic regions exhibiting a certain level of mapping depth is computed for each sample. These data are then represented and compared using a boxplot stratified by DNA source. (PDF) [file pgen.1010625.s002.pdf]

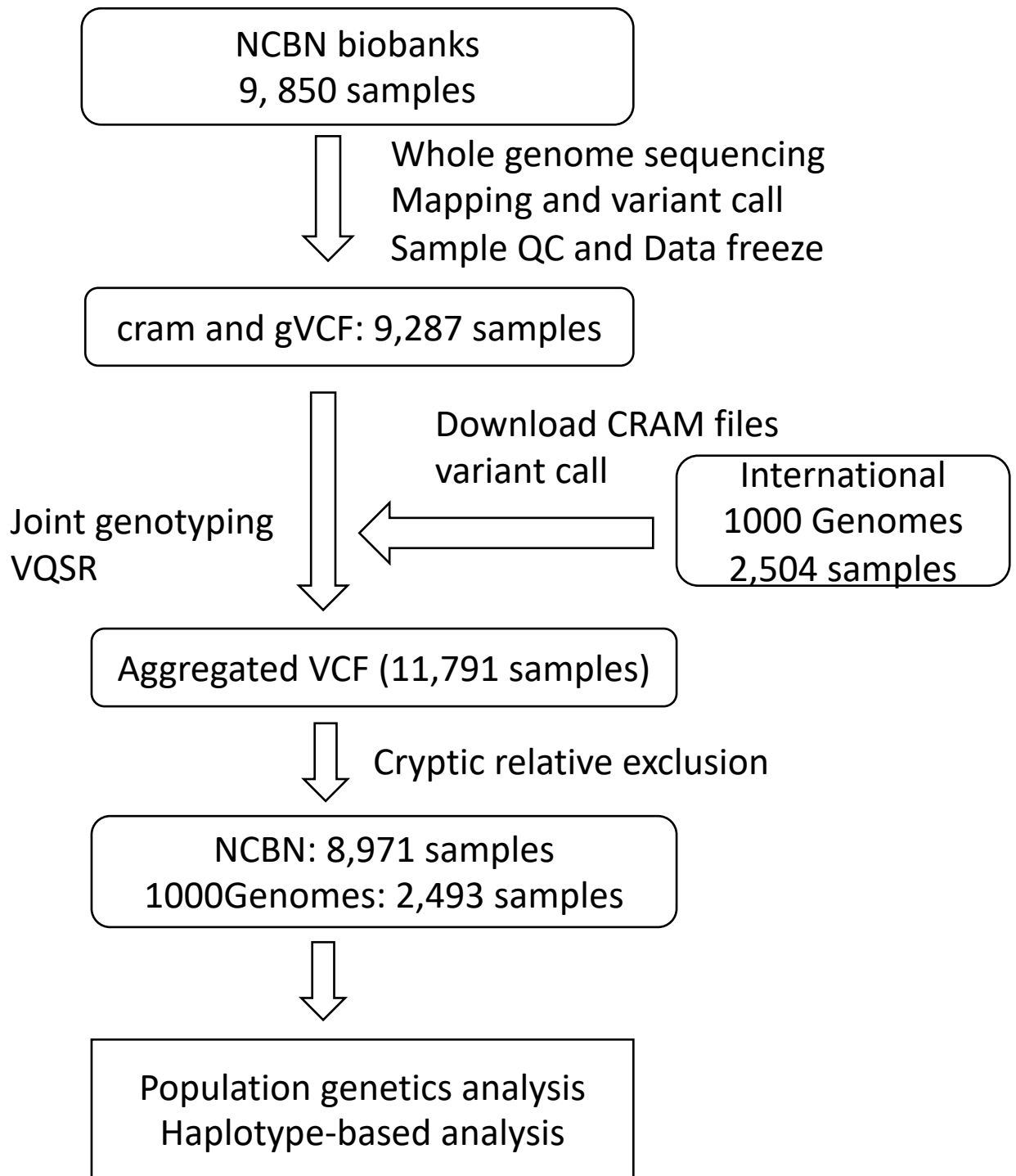

**S3 Fig: Breakdown of QC steps and sample size for this study**

Supplement: S3 Fig — The steps in the analysis and QC process are illustrated in the figure, with a breakdown of the number of samples. (PDF) [file pgen.1010625.s003.pdf]

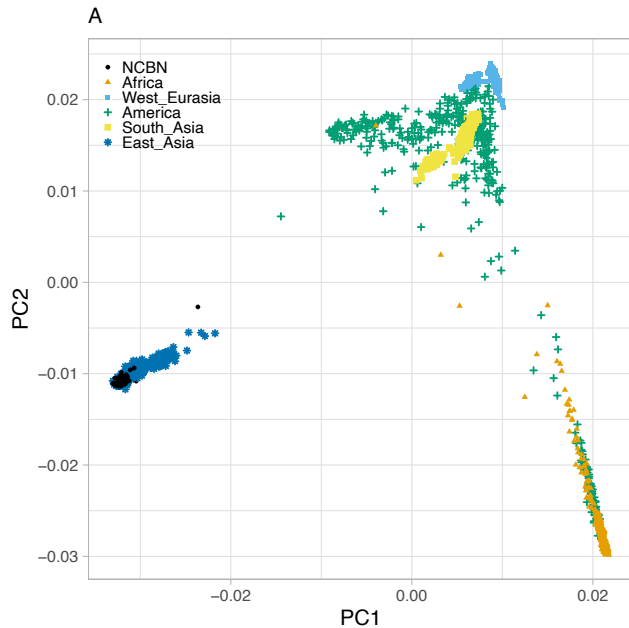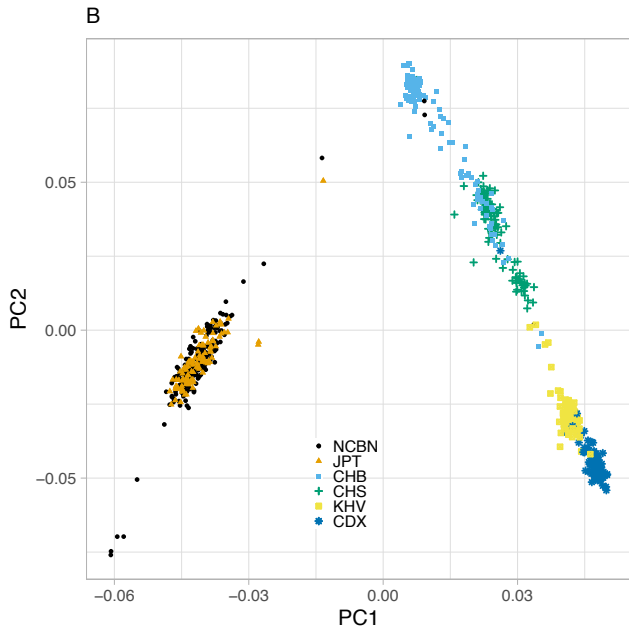

**S4 Fig: PCA performed with the number of NCBN samples reduced to 200**

Supplement: S4 Fig — A PCA was performed on 200 randomly selected NCBN samples under the same conditions as in Fig 2. (A) The first and second principal components are plotted. The continental population of the international 1000 genomes and NCBN are plotted in different colors and shapes. (B) PCA plots of the East Asian population of the International 1000 Genomes and NCBN samples are shown. (PDF) [file pgen.1010625.s004.pdf]

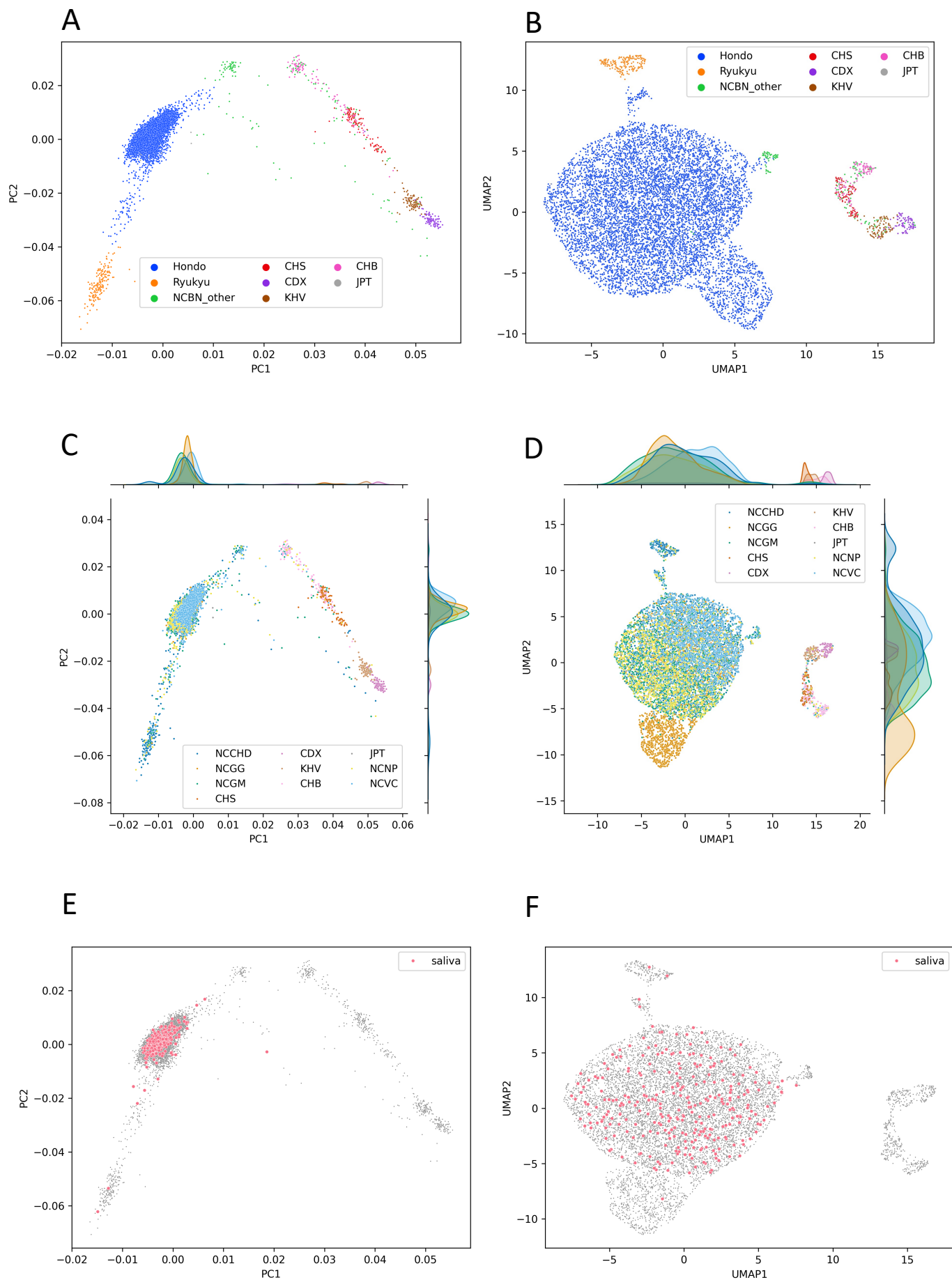

**S5 Fig: Genetic structure of East Asian populations**

Supplement: S5 Fig — (A) The clusters consisting of the NCBN samples in Fig 2 are classified into Hondo (blue), Ryukyu (orange), and others. (B) The six principal component of PCA were dimensionally reduced to a two-dimensional representation via the Uniform Manifold Approximation and Projection (UMAP). The color scheme employed matches that of (A). The UMAP transformation was executed using the UMAP function from the umap-learn package in Python, with the parameters set to n_neighbors = 10 and min_dist = 0.5. (C) (D) Plot of (A) and (B) color-coded by sample donor biobank. Peripheral density distribution is also attached to show the difference in distribution within clusters. (E) (F) Plots (A) and (B) with saliva-derived samples highlighted. (PDF) [file pgen.1010625.s005.pdf]

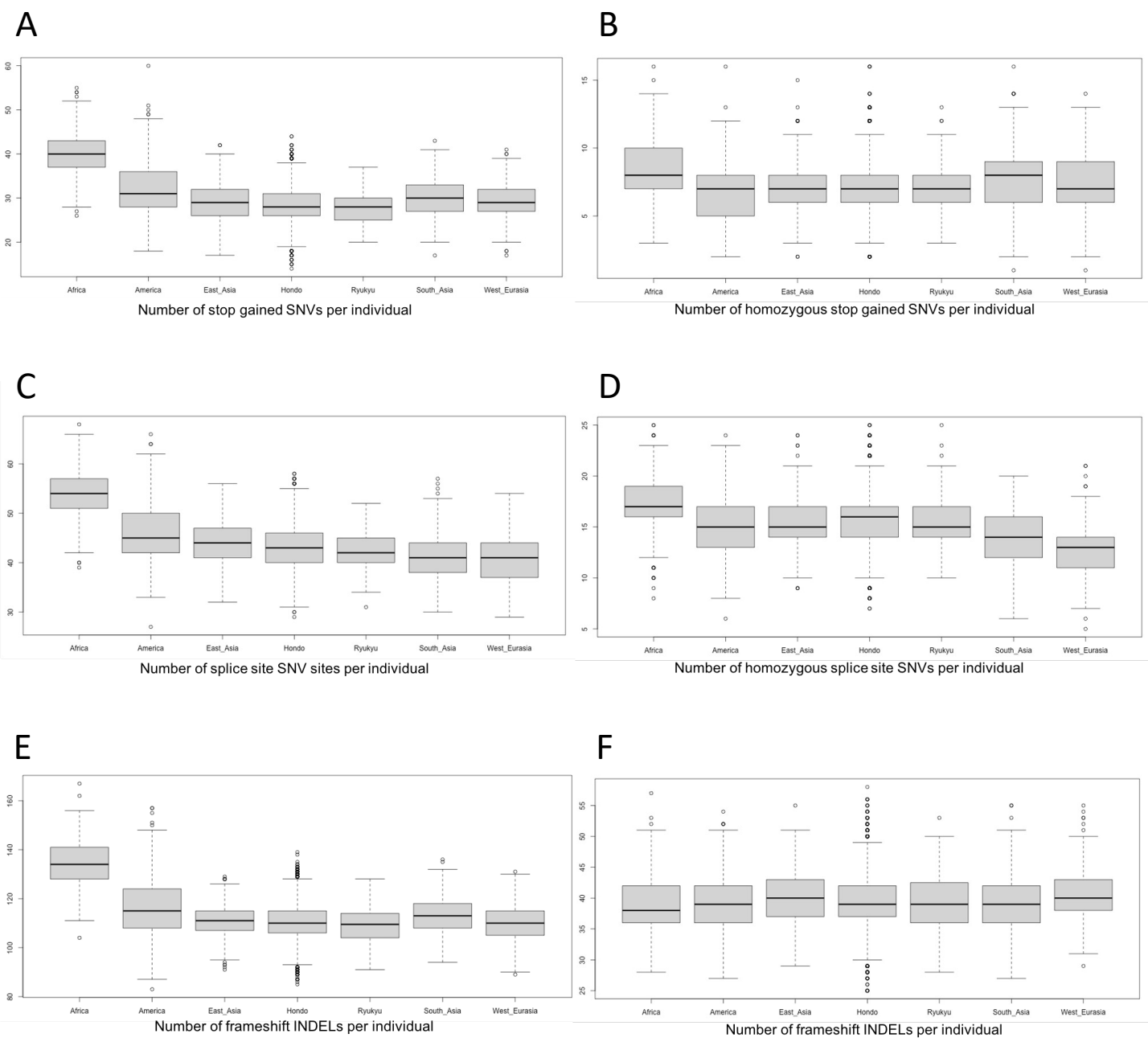

**S6 Fig: Analysis of Loss-of-function (LoF) variants.**

Supplement: S6 Fig — The numbers of LoF sites per individual by category are presented: (A) and (B) stop gained SNV; (C) and (D) splice site SNV; (E) and (F) frameshift INDELs. (PDF) [file pgen.1010625.s006.pdf]

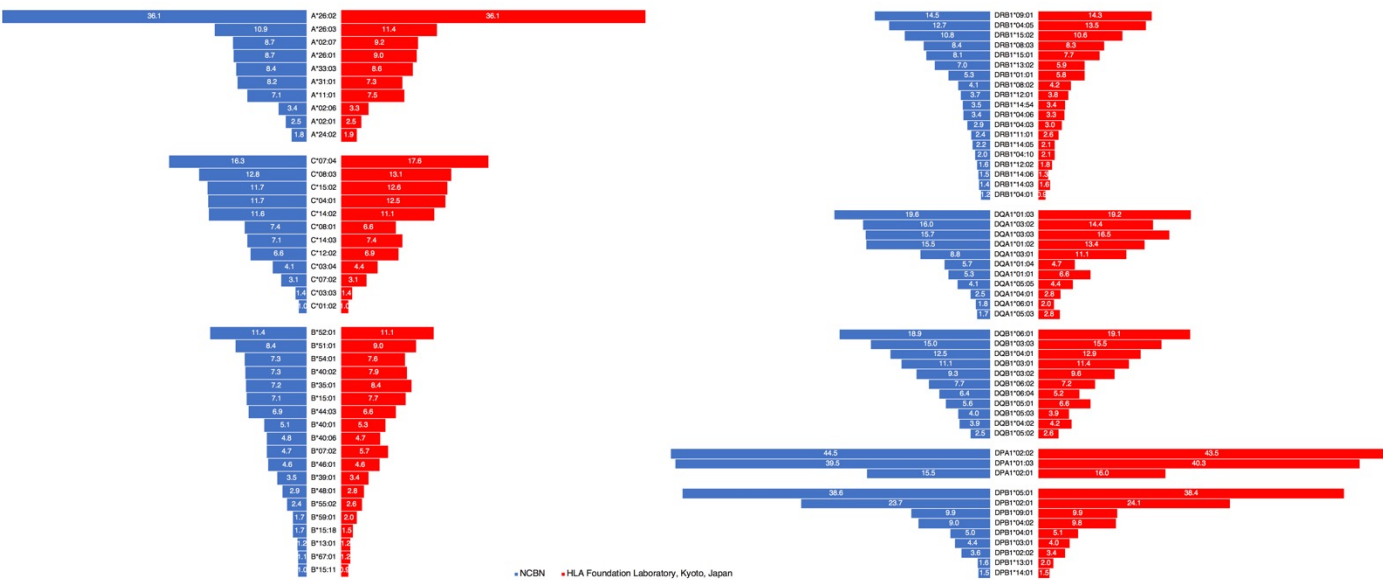

S7 Fig: HLA alleles frequencies (%) between NCBN vs HLA Foundation Laboratory, Kyoto, Japan.

Supplement: S7 Fig — Comparison for class I HLA genes (HLA-A, -C, -B) (left). Comparison for class II HLA genes (HLA-DRB1, -DQA1, -DQB1, -DPA1, -DPB1) (right). Only common HLA alleles (HLA frequencies > 1%) are included in this analysis. (PDF) [file pgen.1010625.s007.pdf]

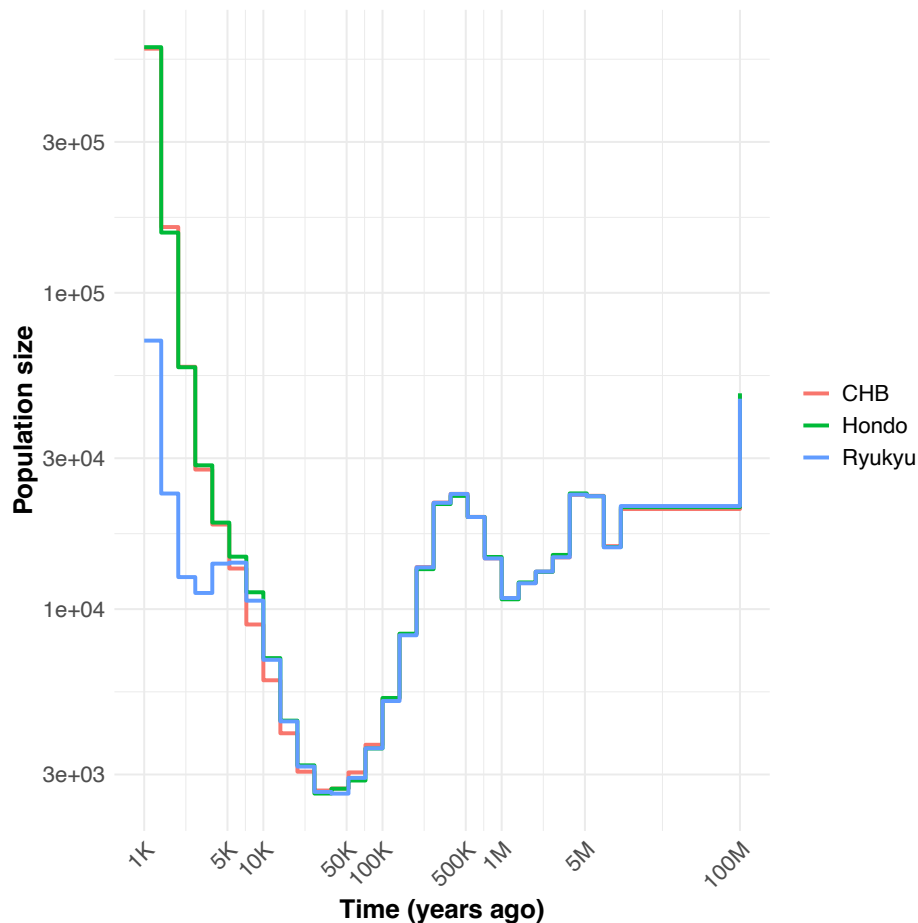

**S8 Fig: Long-term effective population size change of Hondo, Ryukyu and Han Chinese.**

Supplement: S8 Fig — The changes in population size were estimated from the gene genealogy across the genome. (PDF) [file pgen.1010625.s008.pdf]

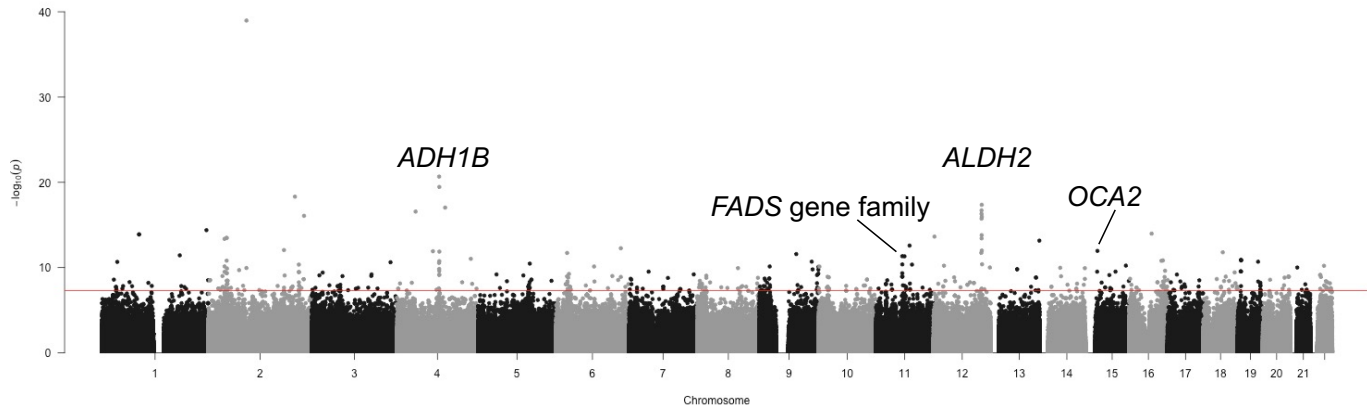

**S9 Fig: Manhattan plot of the selection scan result of the whole genome SNPs by Relate.**

Supplement: S9 Fig — The red line represents the genome-wide significance level (5 × 10^−8^). (PDF) [file pgen.1010625.s009.pdf]

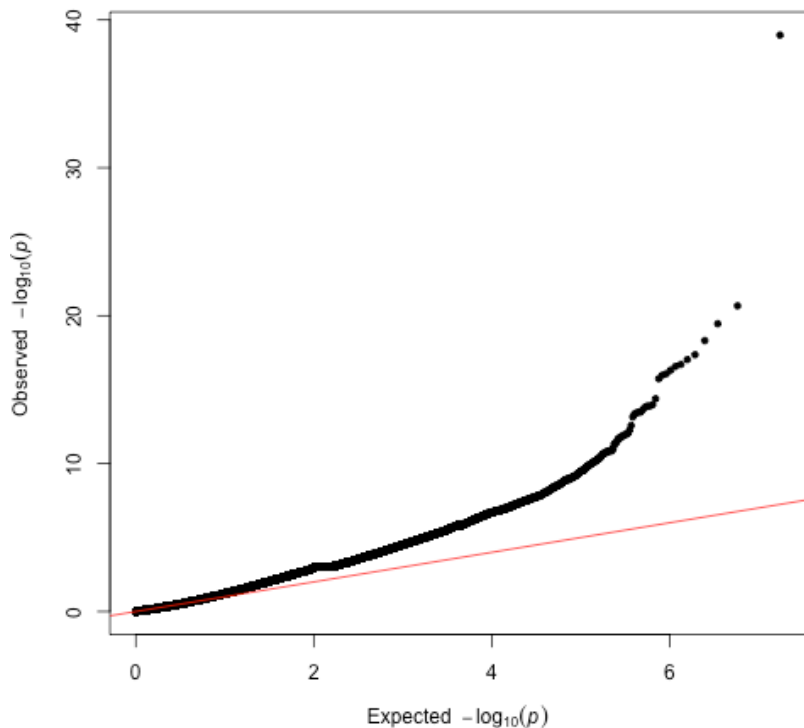

**S10 Fig: QQ plot of the selection scan result of the whole genome SNPs by Relate.**

Supplement: S10 Fig — The red line denotes y = x. (PDF) [file pgen.1010625.s010.pdf]

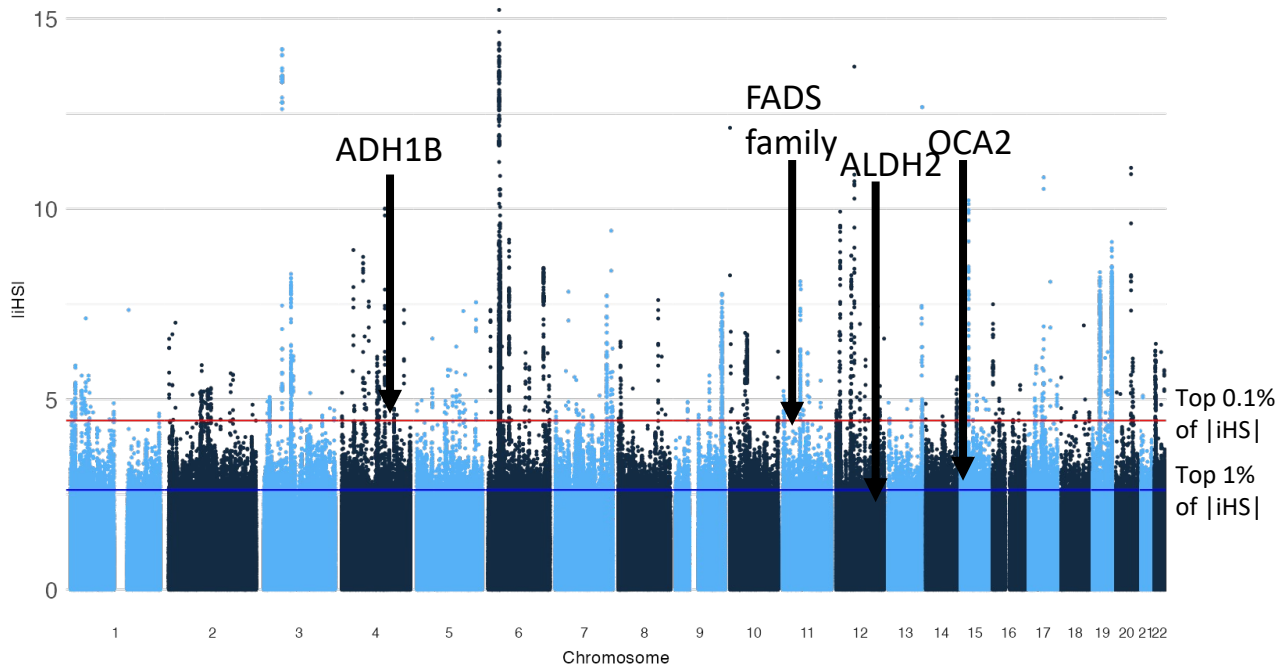

**S12 Fig: Genome-wide signature of positive selection ( $iHS$ ) of the whole genome SNPs by selscan.**

Supplement: S12 Fig — The absolute values of iHS for each SNP are plotted on the vertical axis, with red and blue lines drawn at the top 0.1% and 1% of the genome, respectively, in descending order of absolute iHS value. (PDF) [file pgen.1010625.s012.pdf]
