# Supplementary material for: Exploring the genetic diversity of the Japanese population: Insights from a large-scale whole genome sequencing analysis
Source: PLoS Genet. 2023 Dec 7;19(12):e1010625. doi: 10.1371/journal.pgen.1010625 (PMC10703243; doi:10.1371/journal.pgen.1010625)
Supplement: S11 Fig — Genealogy of (a) ALDH2 rs671, (b) ADH1B rs1229984 (c) OCA2 rs1800414 (d) FADS1 rs174599 are presented. The vertical axis represents the age (years before present). Derived allele carriers are shown in red. (PDF) [file pgen.1010625.s011.pdf]

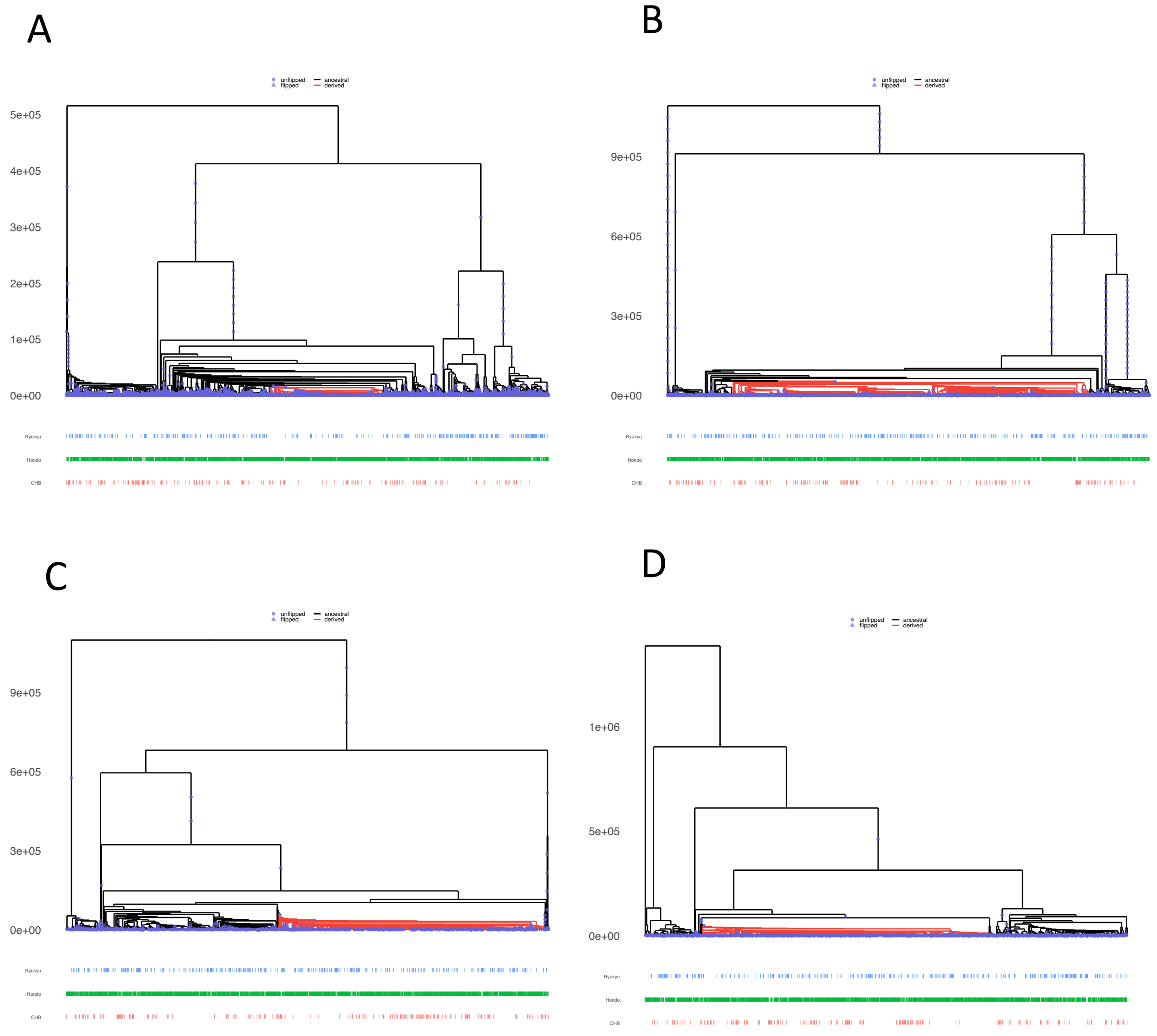

S11 Fig: **Gene genealogy estimated by RELATE.** Genealogy of (a) ALDH2 rs671, (b) ADH1B rs1229984 (c) OCA2 rs1800414 (d) FADS1 rs174599 are presented. The vertical axis represents the age (years before present). Derived allele carriers are shown in red.
