# Supplementary material for: Exploring the genetic diversity of the Japanese population: Insights from a large-scale whole genome sequencing analysis
Source: PLoS Genet. 2023 Dec 7;19(12):e1010625. doi: 10.1371/journal.pgen.1010625 (PMC10703243; doi:10.1371/journal.pgen.1010625)
Supplement: S1 Table — (PDF) [file pgen.1010625.s013.pdf]

Data set	typer of variant	novelty (dbSNP151)	filter(VQSR)	number	$F_{singleton}$	$F_{MAF<0.5\%}$
NCBN+1000G	SNV	known	PASS	106,565,530	41.11%	93.77%
	SNV	known	FAIL	13,086,683	26.93%	92.96%
	SNV	novel	PASS	50,408,517	71.92%	99.99%
	SNV	novel	FAIL	10,197,934	40.16%	99.52%
	INDEL	known	PASS	17,214,879	20.19%	87.42%
	INDEL	known	FAIL	193,890	15.63%	89.05%
	INDEL	novel	PASS	10,675,637	53.53%	99.44%
	INDEL	novel	FAIL	442,789	34.25%	98.84%
	Total			208,785,859	46.49%	95.28%
NCBN	SNV	known	PASS	46,199,619	34.56%	86.73%
	SNV	known	FAIL	7,977,505	23.90%	89.79%
	SNV	novel	PASS	41,046,547	67.46%	99.99%
	SNV	novel	FAIL	8,268,223	36.32%	99.49%
	INDEL	known	PASS	11,122,104	19.71%	82.60%
	INDEL	known	FAIL	149,334	11.12%	87.44%
	INDEL	novel	PASS	7,361,318	47.97%	99.34%
	INDEL	novel	FAIL	334,657	31.30%	98.77%
	Total			122,459,307	44.43%	92.65%

S1 Table. **Summary of variants discovered by WGS.**
