# Supplementary material for: Exploring the genetic diversity of the Japanese population: Insights from a large-scale whole genome sequencing analysis
Source: PLoS Genet. 2023 Dec 7;19(12):e1010625. doi: 10.1371/journal.pgen.1010625 (PMC10703243; doi:10.1371/journal.pgen.1010625)
Supplement: S3 Table — (PDF) [file pgen.1010625.s015.pdf]

rs ID	Gene	P value	Effect size	Tissue
rs174599	FADS2	3.90E-41	0.6	Whole Blood
rs174599	FADS2	1.20E-24	0.44	Esophagus - Muscularis
rs174599	FADS1	2.60E-24	-0.36	Esophagus - Mucosa
rs174599	FADS1	5.00E-24	-0.71	Brain - Cerebellum
rs174599	FADS1	1.50E-22	-0.56	Pancreas
rs174600	FADS2	1.50E-43	0.65	Whole Blood
rs174600	FADS2	7.10E-25	0.29	Cells - Cultured fibroblasts
rs174600	FADS2	3.30E-24	0.47	Esophagus - Muscularis
rs174601	FADS2	4.40E-41	0.6	Whole Blood
rs174601	FADS1	8.50E-25	-0.36	Esophagus - Mucosa
rs174601	FADS2	2.70E-24	0.44	Esophagus - Muscularis
rs174601	FADS1	5.90E-23	-0.7	Brain - Cerebellum
rs174601	FADS1	1.70E-22	-0.56	Pancreas
rs97384	FADS2	4.50E-42	-0.62	Whole Blood
rs97384	FADS1	6.50E-25	0.62	Pancreas
rs97384	FADS1	4.80E-22	0.68	Brain - Cerebellum
rs97384	FADS1	2.40E-21	0.35	Esophagus - Mucosa
rs97384	FADS2	3.10E-21	-0.42	Esophagus - Muscularis

S3 Table. Abbreviated GTEx eQTL Results of SNPs affected by positive natural selection in FADS gene family, P Value Cut Off of 10^{-20}
